# IFNγ-producing CD4^+^ T lymphocytes: the double-edged swords in tuberculosis

**DOI:** 10.1186/s40169-017-0151-8

**Published:** 2017-06-15

**Authors:** Pawan Kumar

**Affiliations:** Special Centre for Molecular Medicine, Jawaharlal Nehru University, New Mehrauli Road, New Delhi, 110067 India

**Keywords:** Tuberculosis, Protection, Pathogenesis, IFN-gamma, CD4^+^ T cell, TB–IRIS, Macrophage, Neutrophil, Necrosis, Matrix metalloproteinase, Granuloma

## Abstract

IFNγ-producing CD4^+^ T cells (IFNγ^+^CD4^+^ T cells) are the key orchestrators of protective immunity against *Mycobacterium tuberculosis* (*Mtb*). Primarily, these cells act by enabling *Mtb*-infected macrophages to enforce phagosome-lysosome fusion, produce reactive nitrogen intermediates (RNIs), and activate autophagy pathways. However, TB is a heterogeneous disease and a host of clinical and experimental findings has also implicated IFNγ^+^CD4^+^ T cells in TB pathogenesis. High frequency of IFNγ^+^CD4^+^ T cells is the most invariable feature of the active disease. Active TB patients mount a heightened IFNγ^+^CD4^+^ T cell response to mycobacterial antigens and demonstrate an IFNγ-inducible transcriptomic signature. IFNγ^+^CD4^+^ T cells have also been shown to mediate TB-associated immune reconstitution inflammatory syndrome (TB–IRIS) observed in a subset of antiretroviral therapy (ART)-treated HIV- and *Mtb*-coinfected people. The pathological face of IFNγ^+^CD4^+^ T cells during mycobacterial infection is further uncovered by studies in the animal model of TB–IRIS and in *Mtb*-infected PD-1^−/−^ mice. This manuscript encompasses the evidence supporting the dual role of IFNγ^+^CD4^+^ T cells during *Mtb* infection and sheds light on immune mechanisms involved in protection versus pathogenesis.

## Background

Tuberculosis (TB) continues to be one of the major causes of morbidity and mortality worldwide [1]. *Mycobacterium tuberculosis* (*Mtb*), the causative agent of TB, mainly resides in host macrophages and modulates their cellular physiology to support its own growth and duplication. Although macrophages are armed with a battery of antimicrobial mechanisms, elimination of the intracellular bacilli from these cells is largely dependent on activation signals arising from CD4^+^ T lymphocytes [2]. A key role in CD4^+^ T cell-mediated macrophage activation can be attributed to IFNγ, which helps in *Mtb* clearance by inducing iNOS expression and enforcing phagosome-lysosome fusion [2]. T_H_1-polarized CD4^+^ T cells are the main source of IFNγ during mycobacterial infections. Significantly enhanced susceptibility to TB in people suffering from HIV/AIDS or inherited IFNγ deficiencies underlines the critical role of IFNγ-producing CD4^+^ T cells (IFNγ^+^CD4^+^ T cells) in host resistance to *Mtb* [2].

The protective role of IFNγ^+^CD4^+^ T cells against *Mtb*, however, is one side of the story. With advancements in TB immunobiology, a host of studies has emerged to show that IFNγ^+^CD4^+^ T cells are directly or indirectly involved in TB pathogenesis. It has been observed that active TB patients mount a heightened T_H_1 type of CD4^+^ T cell responses against *Mtb* antigens [3]. T_H_1 type of CD4^+^ T cell response can also be observed in latently infected people, but its aggravation commonly precedes the reactivation of latent TB into the active disease [3]. Similar aggravation of T_H_1 type of antimycobacterial immunity also occurs in TB–associated immune reconstitution inflammatory syndrome (TB–IRIS) observed in a subset of antiretroviral therapy (ART)-treated HIV- and *Mtb*-coinfected people [4]. Studies in both human patients and the animal models have attributed the pathogenesis of TB–IRIS to hyperactive IFNγ^+^CD4^+^ T cell responses [3, 5].

The present manuscript elaborates on the clinical and experimental findings demonstrating the protective versus the pathological role of IFNγ^+^CD4^+^ T cells during *Mtb* infection. It further sheds light on the underlying mechanisms of IFNγ^+^CD4^+^ T cell-mediated protection against TB and TB pathogenesis. Besides broadening individual’s perspective of TB immunobiology, this manuscript will prompt the TB vaccinologists to retrospect their strategies to combat this age-old disease.

## Protective role of IFNγ^+^CD4^+^ T cells against *Mtb*

Nearly one-third of the world’s population is infected with *Mtb* [6]. However, most of the latently infected people never develop active TB, demonstrating the competence of their immune system to contain the bacilli [2]. Both human and animal studies have established that IFNγ^+^CD4^+^ T cells are the key mediators of protective immunity against *Mtb*. It has been shown that mice deficient in IFNγ are unable to control low-dose *Mtb* infection and succumb to the progressive disease [7–9]. As CD4^+^ T lymphocytes are the most important source of IFNγ during mycobacterial infection, animals deficient in CD4^+^ T cells have also been found to be susceptible to low-dose *Mtb* infection. Other lymphocyte subsets such as CD8^+^ T cells, natural killer (NK) cells, CD1-restricted T cells and γδ T cells also secrete IFNγ in response to *Mtb* infection, but they are unable to compensate for the lack of CD4^+^ T lymphocytes as a source of this cytokine [2]. Mounting of IFNγ^+^CD4^+^ T cell responses relies on IL-12 secretion by antigen-presenting cells. Consistently, animals deficient in IL-12 are also unable to control *Mtb* infection and die of the progressive disease [10, 11].

The importance of IL-12/IFNγ axis in protection against human TB is illustrated by people having mutations in the genes encoding these cytokines [2]. Such people exhibit Mendelian susceptibility to mycobacterial disease (MSMD) and are predisposed to progressive infection with BCG and environmental non-tuberculous mycobacteria [12, 13]. Similarly, lack of IFNγ receptor 1 (IFNγR1) has been shown to cause fatal lepromatoid BCG infection and disseminated non-tuberculous mycobacterial disease [13, 14]. Since IFNγ production depends on IL-12, deficiency in IL-12 receptor β1 (IL-12Rβ1) has also been shown to result in severe primary TB in the affected individuals [2, 15, 16].

One of the most important evidence supporting the protective role of CD4^+^ T cells against human TB is provided by people suffering from HIV/AIDS. Infection with HIV leads to selective deletion of CD4^+^ T lymphocytes, which in turn results in the significantly enhanced susceptibility to TB [17]. Owing to the unrestricted growth of the bacilli, TB frequently affects extrapulmonary sites in HIV/AIDS patients, and can also occur in disseminated form in the severe cases. Similar to the case of HIV/AIDS, idiopathic CD4^+^ T cell lymphocytopenia has also been shown to increase TB susceptibility and its associated mortality [18, 19].

## Mechanisms of IFNγ^+^CD4^+^ T cell-mediated protection against *Mtb*

Despite the long known role of IFNγ^+^CD4^+^ T cells in protection against *Mtb*, its underlying mechanisms are not completely understood. Studies aimed at elucidating the mechanisms of IFNγ-mediated protection against *Mtb* have largely focused on its effect on the infected macrophages. These studies have revealed that IFNγ-activated macrophages eliminate the intracellular bacilli primarily by: (i) producing reactive nitrogen intermediates (RNIs), (ii) enforcing phagosome-lysosome fusion, and (iii) activating the autophagy pathway.

Nitric oxide and other RNIs help in the clearance of the intracellular bacilli by inflicting oxidative damage on to them [20]. *Mtb*-infected macrophages, however, did not produce copious amounts of RNIs in the absence of activating signals. IFNγ promotes iNOS expression in *Mtb*-infected macrophages which catabolizes l-arginine into nitric oxide (NO), which in turn is used as the substrate to generate other RNIs [20, 21]. The indispensable role of RNIs in protection against TB is demonstrated by the enhanced susceptibility of iNOS^−/−^ mice to *Mtb* [22]. Besides having a direct effect on intracellular *Mtb*, RNIs can also reduce the bacillary load by inducing apoptotic cell death in infected macrophages [21]. Apoptosis of infected macrophages is a protective response and is associated with diminished *Mtb* survival. As *Mtb*-containing apoptotic bodies are readily phagocytosed by dendritic cells, it also augments *Mtb*-specific immunity [23].

One of the important strategies evolved by *Mtb* and other pathogenic mycobacteria to survive within infected macrophages is to inhibit phagosome maturation. By excluding vacuolar H^+^-ATPase, pathogenic mycobacteria inhibit phagosome acidification and escape the degradative action of lysosomal acid hydrolases [24]. Studies with *Mtb*-infected macrophages have shown that IFNγ signalling can activate these cells to enforce phagosome maturation and eliminate the intracellular bacilli [25]. Transcription of pH-responsive genes in IFNγ-activated macrophages and attenuation of the acid-susceptible *Mtb* strains in the infected animals shows that IFNγ signalling enables the infected macrophages to overcome phagosomal maturation block both in vitro and in vivo [26, 27].

Autophagy was initially described as a cell survival mechanism during starvation. A plethora of recent studies has demonstrated that autophagy also plays a key role in protection against the intracellular pathogens including *Mtb* [28]. Antimycobacterial effects of autophagy have been attributed to enhanced killing of mycobacteria within the infected cells and reduced inflammation in the affected tissues [29, 30]. The genetic link between autophagy, inflammatory conditions, and TB susceptibility provides an important support to the role of anti-inflammatory and bactericidal properties of autophagy in protection against human TB [31, 32]. IFNγ is a potent autophagy inducer in the *Mtb*-infected macrophages and produces the cellular effects similar to that of starvation [33]. Studies by Matsuzawa et al. have shown that IFNγ-induced macrophage autophagy is mediated by JAK1/2, PI3K, and p38MAPK and is independent of STAT1 [34]. Interestingly, T_H_2 cytokines IL-4 and IL-13 have been shown to hamper IFNγ-induced autophagy in macrophages, suggesting an alternate mechanism for their deleterious effects on anti-*Mtb* immunity [35].

## Pathological role of IFNγ^+^CD4^+^ T cells during *Mtb* infection

Despite their protective role against *Mtb*, IFNγ^+^CD4^+^ T cells have been implicated in TB pathogenesis by a number of studies. This section presents the clinical and experimental findings demonstrating the involvement of IFNγ^+^CD4^+^ T cells in TB pathogenesis. Notably, the pathological character of IFNγ^+^CD4^+^ T cells is predominantly manifested in a subset of *Mtb*-infected immunocompetent adults and TB–IRIS patients, wherein these cells exhibit excessive responsiveness to mycobacterial antigens [3]. In immunocompetent adults, active TB develops from the reactivation of latent infection and primarily affects lung tissue. An overly intense IFNγ^+^CD4^+^ T cell response is the most important immunological parameter distinguishing the active disease from latent infection in these people [27]. On the contrary, the incompetence of host immune system to contain the bacilli is responsible for TB pathogenesis in young children and immunodeficient people [3].

Classical evidence supporting the pathological role of IFNγ^+^CD4^+^ T cells during *Mtb* infection is provided by tuberculin skin testing (TST)—a diagnostic test to examine *Mtb* exposure. TST involves the intradermal injection of purified *Mtb* antigens, followed by monitoring for the delayed-type hypersensitivity (DTH) reaction seen as local skin induration. As DTH is mediated by T_H_1-polarized CD4^+^ T cells, a larger area of skin induration in TB patients demonstrates a strong association between IFNγ^+^CD4^+^ T cells and the active disease [36]. In latently infected people, the area of skin induration has been found to correlate with the future risk of active TB [37]. Therefore, an intense tuberculin reaction is considered as more serious and indicates the likelihood of the concomitant active disease or its future risk [38]. However, it should be noted that lack of reactivity or anergy to *Mtb* antigens does not predict the resistance to active TB. As it signifies the lack of T_H_1 response to mycobacteria, anergy to mycobacterial antigens is associated with enhanced risk of morbidity and mortality in the infected people [39].

Studies aimed at characterizing the host immune response during *Mtb* infection further suggest the involvement of IFNγ^+^CD4^+^ T cells in TB pathogenesis. These studies have shown the heightened levels of IFNγ in lung tissue, broncho-alveolar lavage (BAL) fluid, pleural effusion, and lymph nodes of active TB patient [2]. BAL fluid IFNγ levels in active TB patients have been found to directly correlate with the disease severity and subsided with its successful treatment [40]. These findings are supported by transcriptomic analysis of whole blood cells from TB patients and healthy controls by Berry et al. [41]. The authors have observed an enhanced transcription of IFNγ-inducible genes in active TB patients, compared with latently infected people and healthy controls. Consistent with enhanced IFNγ levels, increased frequency of IFNγ-producing CD4^+^ T cells during active TB has been reported by several studies [42–45]. Although most of these studies have shown the polyfunctionality of CD4^+^ T cells in active TB patients, the specific role of IFNγ^+^CD4^+^ T cells in TB pathogenesis is evident from animal studies.

Unequivocal support for the pathological role of IFNγ-producing CD4^+^ T cells during *Mtb* infection is provided by TB–associated immune reconstitution inflammatory syndrome (TB–IRIS). Affecting a subset of antiretroviral therapy (ART)-treated HIV- and *Mtb*-coinfected people, TB–IRIS occurs in diverse manifestations and poses a major challenge in the clinical management of HIV in these people. Mechanistically, the ART-mediated decline in the viral load allows for the rapid expansion of *Mtb*-specific CD4^+^ T cells [4, 46]. Both terminally-differentiated and effector memory CD4^+^ T cells with specificity to *Mtb* antigens have been shown to expand in ART-treated HIV- and *Mtb*-coinfected people [47, 48]. Exaggeration of antimycobacterial immunity (with CD4^+^ T cell expansion) in the ART-treated HIV- and *Mtb*-coinfected people is evidenced by their conversion from a ‘negative’ TST status to a strongly ‘positive’ one [49]. Interestingly, ART-treated people who develop TB–IRIS demonstrate a more strong T_H_1 type of CD4^+^ T cell response to *Mtb* antigens, compared with those who do not experience this condition [46, 50]. Evidently, immunological parameters in TB–IRIS patients pint out an active participation of IFNγ^+^CD4^+^ T cells in TB pathogenesis.

Direct involvement of IFNγ^+^CD4^+^ T cells in TB–IRIS development is confirmed by a mouse model, wherein the human disease has been mimicked by adoptively transferring naïve CD4^+^ T cells into *M. avium*-infected, T cell-deficient (TCRα^−/−^) mice [5]. It has been shown that adoptively transferred CD4^+^ T lymphocytes rapidly acquired T_H_1 phenotype and led to the failure of lung function, wasting and eventual death of host animals. The authors have further noted that ability of the donor lymphocytes to cause lung pathology was lost in IFNγ-deficient CD4^+^ T cells [5]. Thus, both human and animal studies attribute the pathogenesis of TB–IRIS to *Mtb*-specific IFNγ^+^CD4^+^ T cells.

Studies in PD-1^−/−^ mice and a macaque model further confirm the direct involvement of IFNγ^+^CD4^+^ T cells in TB pathogenesis [51]. PD-1 is present on T lymphocytes and its engagement by PD-L1 results in the negative regulation of T cell functions [2]. As PD-L1 was found to be abundant in TB patients, researchers wondered over the outcome of PD-1 signalling during *Mtb* infection and examined it using knockout mouse strains [51, 52]. Surprisingly, it was observed that instead of developing resistance to *Mtb*, PD-1^−/−^ mice exhibited significantly enhanced susceptibility to mycobacterial infection. Further analysis of TB pathogenesis in PD-1^−/−^ mice revealed that these animals mount an exaggerated IFNγ^+^CD4^+^ T cell response to the bacilli [51]. Besides PD-1, CD4^+^CD25^+^FoxP3^+^ T regulatory (T_reg_) cells can also suppress the exaggerated IFNγ^+^CD4^+^ T cell response during mycobacterial infection [53, 54]. In macaque model of TB, higher frequency of T_reg_ cells has been observed in the animals who would develop the latent infection, compared with those that would develop the active disease [55]. These findings in PD-1^−/−^ mice and the macaque model demonstrate that aggravated IFNγ^+^CD4^+^ T cell response is the key mediator of TB pathology and that its inhibition prevents the reactivation of latent infection into the active disease.

**Fig. 1:**
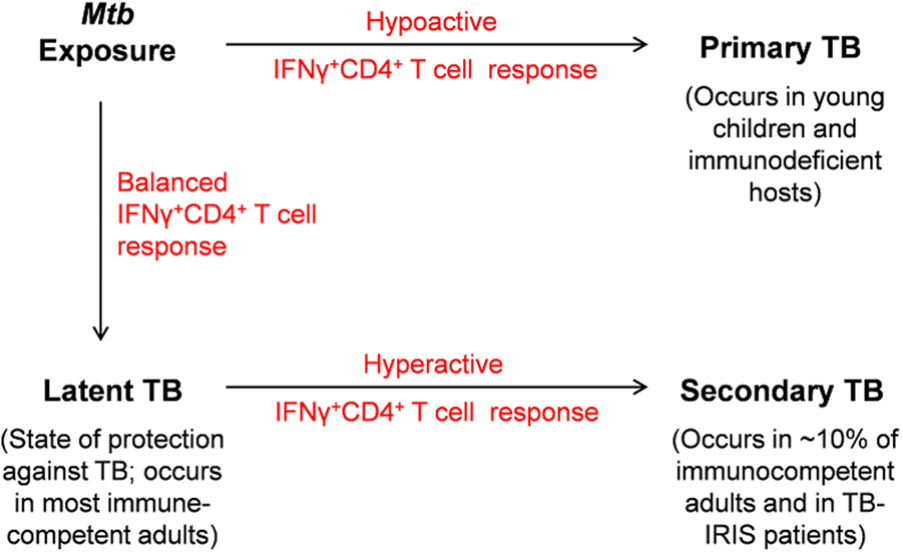
Protective versus pathological role of IFNγ^+^CD4^+^ T cells during *Mycobacterium tuberculosis* (*Mtb*) infection. *Mtb* exposure in most young children and immunodeficient people evokes a hypoactive IFNγ^+^CD4^+^ T cell response, which is inefficient in containing the bacilli. Therefore, *Mtb* infection in these people results in primary TB, frequently affecting the extrapulmonary sites. In contrast, most of the immunocompetent adults mount a balanced immune response to *Mtb* and contain it in the form of latent TB. With the course of time, IFNγ^+^CD4^+^ T cell response would aggravate in nearly 10% of latently infected people, leading to the development of active TB in them. Similar aggravation of IFNγ^+^CD4^+^ T cells is to blame for TB–IRIS development in a subset of ART-treated, HIV- and *Mtb*-coinfected people

It is evident from the above-discussed findings that protective versus pathological character of IFNγ^+^CD4^+^ T cells is defined by the degree of their responsiveness to *Mtb*. A hyperactive IFNγ^+^CD4^+^ T cell response to *Mtb* is pathological in nature and is frequently observed in adult TB patients. After initial exposure, most of the immunocompetent adults would contain *Mtb* infection without developing any disease symptom. This condition of asymptomatic *Mtb* infection, referred to as latent TB, represents the state of protection against the bacilli. Latent TB persists lifelong in most, but nearly 10% of the infected people who would develop active TB in their lifetime. As discussed above, reactivation of latent infection into the active disease can be attributed to aggravated anti-*Mtb* IFNγ^+^CD4^+^ T cell responses. Similar aggravation of *Mtb*-specific IFNγ^+^CD4^+^ T cell responses is to blame for TB–IRIS development in a subset of HIV- and *Mtb*-coinfected people. In contrast to immunocompetent adults and TB–IRIS patients, most young children and immunodeficient people mount a hypoactive IFNγ^+^CD4^+^ T cell response to *Mtb*, which is inefficient in containing the bacilli. Therefore, *Mtb* infection in these people leads to primary TB, frequently affecting the extrapulmonary sites. The protective versus pathological role of IFNγ^+^CD4^+^ T cells during *Mtb* infection is summarized in Fig. 1.

## Mechanisms of IFNγ^+^CD4^+^ T cell-mediated TB pathogenesis

Preferential expression of IFNγ-inducible genes in neutrophils (and to some extent in monocytes) during active TB indicates the involvement of these cells in IFNγ-mediated TB pathogenesis [56]. Neutrophils are frequently infected by *Mtb* and are abundant at the site of active disease. Although these cells may help in the containment of the bacilli during the initial phase of infection, their involvement in TB pathogenesis is supported by a number of studies. It has been shown that the frequency of neutrophils at the site of active disease correlates with the disease severity [57]. Higher neutrophil count (neutrophilia) is associated with low sputum conversion and poor TB prognosis [57, 58]. Increased frequency of neutrophils at the affected site has also been demonstrated in susceptible mouse strains and their depletion from these animals resulted in the enhanced resistance to *Mtb* [59]. Interestingly, IFNγ has been shown to increase neutrophil lifespan which may potentially contribute to neutrophilia in the infected animals. Besides, the functional activity of neutrophils is also bolstered by IFNγ [60].

*Mtb*-infected neutrophils and macrophages are the potent producers of toxic molecules and matrix degrading enzymes, including elastases, myeloperoxidases, collagenases, and serine proteases. MMP-1 is a key collagenase up-regulated in TB patients and its enhanced levels have been shown to be associated with increased lung pathology in a transgenic mouse model [61]. MMP-9, which has been implicated in the pathogenesis of many inflammatory diseases, is also abundant in active TB patients and is associated with poor prognosis of the disease [62]. Interestingly, a heightened IFNγ^+^CD4^+^ T cell response has been shown to be associated with enhanced MMP production [63]. Additionally, IFNγ^+^CD4^+^ T cell-activated neutrophils and macrophages produce copious amounts of reactive nitrogen intermediates (RNIs) and reactive oxygen species (ROS) which can damage the healthy tissue [64]. A combined action of tissue-digesting enzymes and RNI/ROS can result in the dismantling of granuloma and progression of latent infection into active TB. Supporting this, higher neutrophil and inflammatory monocyte frequency, elevated serum nitrate levels, and enhanced MMP expression have been observed in the *Mtb*-infected animals, wherein pathology was mediated by IFNγ^+^CD4^+^ T cells [5, 63].

Necrotic cell death of neutrophils and macrophages also plays a key role in TB pathogenesis. The pathological role of necrotic cell death in TB has been demonstrated elegantly in the zebrafish model, wherein increased production of LXA4 (an inducer of necrosis) resulted in reduced host resistance to mycobacterial infection [65]. These findings are relevant in human TB, for polymorphisms in *Alox5* and *Ita4h*, which regulate necrosis versus apoptosis, has been shown to define TB susceptibility [65, 66]. Although IFNγ promotes the necrosis in *Mtb*-infected macrophages [67], its effect on neutrophils is not clear. It is probable that increased oxidative stress in the presence of IFNγ could direct *Mtb*-infected neutrophils to the necrotic pathway.

Another interesting mechanism of IFNγ-mediated TB pathology has been demonstrated by Aly and co-workers [68]. The authors have demonstrated that by altering the balance between angiostatic and angiogenic mediators, IFNγ disrupts the granuloma vascularization and leads to a hypoxic central core. Deprived of nutrients and oxygen supply, the core of granuloma necrotizes and undergoes caseation, resulting in the activation of latent infection into active TB [68]. It is likely that the combined action of this and above-discussed mechanisms leads to IFNγ^+^CD4^+^ T cell-mediated TB pathogenesis in immunocompetent adults. The individual contribution of these mechanisms to the development of TB, however, awaits further analysis.

## Conclusion and future perspectives

IFNγ^+^CD4^+^ T cells are the key orchestrators of antimycobacterial immunity. However, with increasing recognition of TB as a heterogeneous disease [2], IFNγ^+^CD4^+^ T cells have also been implicated in TB pathogenesis. A more intense tuberculin reaction, which is driven by IFNγ^+^CD4^+^ T cells, is frequently observed in TB patients, and its intensity in latently infected people indicates the future risk of the active disease in them [27]. Active TB patients exhibit enhanced IFNγ levels in different tissues which correlate with disease severity [2, 40]. In keeping with this, increased expression of IFNγ-inducible genes has been observed in active TB patients, compared with latently infected people and healthy controls [56]. The pathological face of IFNγ^+^CD4^+^ T cells during *Mtb* infection is also obvious in TB–IRIS patients and its animal model [5]. Enhanced susceptibility of PD-1^−/−^ mouse strains, which mount heightened IFNγ^+^CD4^+^ T cell responses to *Mtb*, further support the pathological role of these cells during *Mtb* infection [51]. Disrupted granuloma vascularization and/or neutrophil- and macrophage-mediated dismantling of granuloma architecture are important mechanisms of IFNγ^+^CD4^+^ T cell-mediated TB pathology.

Owing to earlier studies demonstrating the protective role of IFNγ^+^CD4^+^ T cells during *Mtb* infection, vaccinologists had been aiming to boost IFNγ^+^CD4^+^ T cell response as a strategy to TB immunoprophylaxis. Unfortunately, these strategies have failed to confer significant protection against human TB [69]. Failure of rationally designed vaccines against human TB and the dual role of IFNγ^+^CD4^+^ T cells during *Mtb* infection call for retrospection of our approach to TB vaccination. It is imperative that instead of boosting antimycobacterial immunity, researchers must attempt to dampen the IFNγ^+^CD4^+^ T cell responses in susceptible immunocompetent adults to prevent TB in them. Likely success of these approaches against human TB is suggested by animal studies.

Conclusively, a significant volume of scientific data demonstrates that, besides conferring protection against *Mtb*, IFNγ^+^CD4^+^ T cells also play a key role in TB pathogenesis in immunocompetent adults. As adult TB represents the major burden of the disease, there is an urgency of legitimate efforts to evaluate the immune-dampening approaches in these patients. Not only these approaches can improve treatment outcome against the active disease, they are also likely to help in the effective management of drug-resistant TB, which has emerged as a major challenge for the clinicians.

## Abbreviations

TB: tuberculosis
*Mtb*: *Mycobacterium tuberculosis*
IFN: interferon
TB–IRIS: tuberculosis–associated immune reconstitution inflammatory syndrome
PD-1: programmed cell death protein-1
iNOS: inducible nitric oxide synthase
RNIs: reactive nitrogen intermediates
MMP: matrix metalloproteinase
